# Modulation of epileptic activity by deep brain stimulation: a model-based study of frequency-dependent effects

**DOI:** 10.3389/fncom.2013.00094

**Published:** 2013-07-16

**Authors:** Faten Mina, Pascal Benquet, Anca Pasnicu, Arnaud Biraben, Fabrice Wendling

**Affiliations:** ^1^INSERM, U1099, Universite de Rennes 1Rennes, France; ^2^Laboratoire Traitement du Signal et de L'Image, Université de Rennes 1Rennes, France; ^3^Unité d'Épileptologie, Service de Neurologie, CHURennes, France

**Keywords:** DBS, thalamocortical model, computational, centromedian nucleus, FCD, premotor cortex, epilepsy

## Abstract

A number of studies showed that deep brain stimulation (DBS) can modulate the activity in the epileptic brain and that a decrease of seizures can be achieved in “responding” patients. In most of these studies, the choice of stimulation parameters is critical to obtain desired clinical effects. In particular, the stimulation frequency is a key parameter that is difficult to tune. A reason is that our knowledge about the frequency-dependant mechanisms according to which DBS indirectly impacts the dynamics of pathological neuronal systems located in the neocortex is still limited. We address this issue using both computational modeling and intracerebral EEG (iEEG) data. We developed a macroscopic (neural mass) model of the thalamocortical network. In line with already-existing models, it includes interconnected neocortical pyramidal cells and interneurons, thalamocortical cells and reticular neurons. The novelty was to introduce, in the thalamic compartment, the biophysical effects of direct stimulation. Regarding clinical data, we used a quite unique data set recorded in a patient (drug-resistant epilepsy) with a focal cortical dysplasia (FCD). In this patient, DBS strongly reduced the sustained epileptic activity of the FCD for low-frequency (LFS, < 2 Hz) and high-frequency stimulation (HFS, > 70 Hz) while intermediate-frequency stimulation (IFS, around 50 Hz) had no effect. Signal processing, clustering, and optimization techniques allowed us to identify the necessary conditions for reproducing, in the model, the observed frequency-dependent stimulation effects. Key elements which explain the suppression of epileptic activity in the FCD include: (a) feed-forward inhibition and synaptic short-term depression of thalamocortical connections at LFS, and (b) inhibition of the thalamic output at HFS. Conversely, modeling results indicate that IFS favors thalamic oscillations and entrains epileptic dynamics.

## Introduction

Deep brain stimulation (DBS) for Parkinson's disease (PD) and other movement and psychiatric disorders—including dystonia, tremor, and depression—is clinically used today as a conventional therapeutic procedure for the alleviation of symptoms (Sillay and Starr, 2009). Since the early 90s, neurologists also attempted to apply DBS to other neurological disorders, typically to intractable epilepsies in order to suppress—or at least dramatically reduce—the occurrence of seizures [see recent review in Boon et al. (2009)]. These studies followed early scientific evidence showing potentially beneficial effects of DBS on epileptic neural dynamics in animal models (Reimer et al., 1967; Hablitz, 1976) as well as in patients (Cooper et al., 1973; Davis et al., 1982; Wright and Weller, 1983). However, contrary to PD, the optimal “antiepileptic parameters” of DBS for reducing the frequency of seizures are much more variable among patients and the number of non-responders to stimulation still perplexes scientists. Moreover, in responding patients, the fine tuning of stimulation parameters in a patient-specific manner remains indispensable for maximizing antiepileptic effects. On that account, many fundamental questions are frequently raised: where and when to stimulate, at which frequency, at which current intensity, and with which current waveform?

The answers to these questions remain bound to our current, and still limited, understanding of the mechanisms by which DBS modulates neuronal dynamics, whether normal or pathological. Today, the precise mechanisms of neuronal modulation by DBS remain elusive. In addition, these mechanisms are controversial as observed effects are sometimes opposite (McIntyre et al., 2004b). Among the many studies reported over the last decade, identified mechanisms regarding HFS include: local depolarization blockade by HFS (Beurrier et al., 2001), synaptic depression due to neurotransmitter depletion (Shen et al., 2003; Kim et al., 2012), synaptic inhibition (Filali et al., 2004), disruption of the thalamocortical network's dysrhythmia (McIntyre and Hahn, 2010; Kendall et al., 2011). As far as LFS is concerned, some studies described a transient synaptic depression that alters synaptic transmission (Jiang et al., 2003; Speechley et al., 2007). Finally, IFS is routinely used in the context of presurgical evaluation of patients with drug resistant epilepsy to map epileptogenic and functional brain areas. It has long been observed that this type of stimulation is prone to trigger epileptic afterdischarges (Goddard, 1967). This brief overview shows that the spectrum of involved mechanisms is very large and that distinct stimulation frequencies trigger distinct cellular/network processes. More precise insights into these processes will come with increased knowledge about both biophysical and neurophysiological effects of stimulation currents on underlying neuronal systems.

However, the access to cellular and network mechanisms induced by DBS is rather difficult in animal models of epilepsy and (almost) impossible in patients especially in large-scale systems like the thalamocortical loop. An alternative approach is the use of computational models based on physiological data to first reproduce and then explain changes in cerebral activity as a function of stimulation conditions (stimulation site, intensity, and frequency). This is precisely the objective of this study, with a special focus on the distinct effects of DBS frequency on cortical epileptic dynamics.

Our investigation combines computational modeling and clinical data. We explored stimulation effects in a lumped-parameter mesoscopic neural mass model of the thalamacortical loop, inspired from previously published models (Suffczynski et al., 2004; Lopes Da Silva, 2006; Roberts and Robinson, 2008; Crunelli et al., 2011).

Although these models are lumped representations of underlying neuronal systems, they offer a number of advantages in the context of this study. First, neural mass models include subpopulations of principal excitatory cells and inhibitory interneurons. Second, these models were shown to produce realistic activity as observed in LFPs or EEG under normal (Freeman, 1973; Lopes Da Silva et al., 1974) or epileptic conditions [review in Lytton (2008); Wendling (2008)]. Third, main parameters (mean membrane potential and firing rate) provide access to the investigation of several stimulation-induced (patho)physiological mechanisms. For instance, a neural mass model was successfully used in the context of direct low-intensity pulse stimulation in the hippocampus to explain the behavior of evoked responses during the transition to seizures (Suffczynski et al., 2008).

In particular, using this model, we analyzed the neurophysiological effects induced by direct thalamic stimulation on epileptic cortical dynamics at low frequency (LF, < 20 Hz), intermediate frequency (IF, 20–70 Hz) and high frequency (HF, 70–130 Hz). Model parameters were tuned to reproduce a typical pathological oscillatory activity observed in a neocortical lesion (focal cortical dysplasia, or FCD) in a patient with drug-resistant epilepsy. Intracerebral EEG (iEEG) signals observed during thalamic stimulation (centromedian nucleus) of this patient revealed particularly pronounced frequency-dependent modulation of the FCD pathological activity. Therefore, this data set offered the unique opportunity to identify key model parameters for which such a frequency-dependent modulation could be reproduced and, subsequently to get insights regarding the mechanisms underlying the modulatory effects, in the FCD, of thalamic stimulation. Results revealed that LFS favors feed-forward inhibition and short-term depression at the cortical level and that HFS inhibits the thalamic activity, while IFS reinforces reticulothalamic oscillations thus entraining cortical pathological epileptic dynamics.

## Materials and methods

In this section, we present (1) the neurophysiologically-relevant computational model that we developed to study thalamic DBS, (2) the real depth-EEG dataset used for model tuning and, (3) the signal processing methods used for characterizing real and simulated EEG signals.

### Model of the thalamocortical loop

In order to study the effects of thalamic DBS on cortical dynamics, we implemented a physiologically-plausible mesoscopic model of the thalamocortical loop. This model accounts for the average activity of both cortical and thalamic compartments which include various types of neuronal populations interacting via synaptic transmission. This modeling approach was first proposed in the early 70s (Wilson and Cowan, 1972) and further enriched in order to interpret electrophysiological recordings and study brain dynamics, in the olfactory (Freeman, 1973) and the thalamocortical (Lopes Da Silva et al., 1974) system, for instance, as well as the dynamics of cortical oscillations (Nunez, 1974). This approach was then developed by other research groups in the context of state changes in brain dynamics (Wright et al., 1985), visual evoked potentials (Jansen et al., 1993), dynamics of the human alpha rhythm (Stam et al., 1999) or pathophysiological mechanisms of ictal transitions in epilepsy (Wendling et al., 2000, 2002; Suffczynski et al., 2001; Robinson et al., 2002; Liley and Bojak, 2005; Breakspear et al., 2006). Later, neural mass models were also used in studies dealing with the connectivity among cortical regions and the impact of model parameters on the power spectrum of EEG or MEG signals (Robinson et al., 1997; David and Friston, 2003; Zavaglia et al., 2006).

#### Model architecture

**Figure 1:**
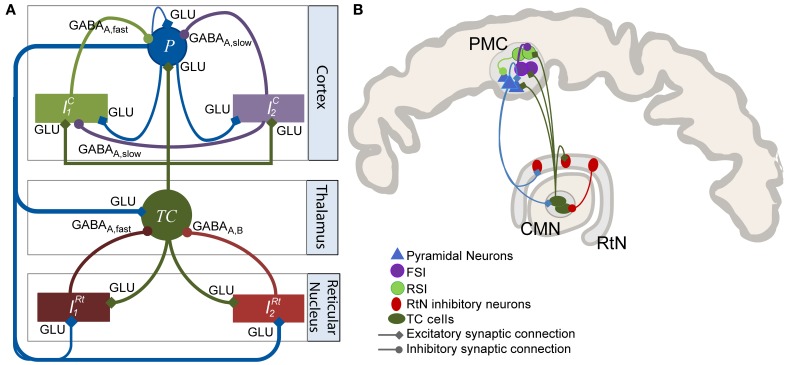
**Model of the thalamocortical loop. (A)** The model architecture comprises three main compartments: cortical, thalamic, and reticular. The cortical compartment includes three subpopulations: P (pyramidal principal neurons), *I^C^*_1_ (soma- and proximal-dendrite targeting interneurons mediating GABA*_A, fast_* currents), and *I^C^*_2_, (dendrite-targeting interneurons mediating GABA*_A, slow_* currents). The thalamic compartment represents a generic thalamic nucleus including a subpopulation of excitatory thalamocortical (*TC*) cells. The reticular nucleus (RtN) compartment is made up of two GABAergic neuronal populations (*I*^*Rt*^_1_, GABA*_A, fast_* currents and *I^Rt^*_2_, GABA*_A, slow_*). Excitatory synaptic transmission among the considered subpopulations is glutamatergic (GLU). **(B)** *Anatomical connectivity of the CMN, PMC, and RtN*. This diagram represents the anatomy of a particular thalamocortical loop interconnecting the CM thalamic nucleus, the PMC, and the RtN. Connectivity patterns were inferred from the literature. It is compatible with the thalamocortical model diagram presented in **(A)**.

The model architecture was inspired from previously published models of the thalamocortical loop (Suffczynski et al., 2004; Lopes Da Silva, 2006; Roberts and Robinson, 2008; Crunelli et al., 2011). In a global view, the model was built of three interconnected compartments: a cortical compartment, a thalamic compartment, and a reticular compartment, in accordance with previously published models (Figure 1A) and with anatomical data (Figure 1B). Each compartment includes one or several subpopulation(s) of neurons, either excitatory or inhibitory. Generally speaking, the input/output functions of a considered subpopulation are represented by two mathematical equations that were respectively named “pulse-to-wave” (input) and “wave-to-pulse” (output) by Walter Freeman (Freeman, 1992). The former is a linear transfer function that converts the presynaptic average density of afferent action potentials into an average postsynaptic membrane potential (PSP), either excitatory (EPSP) or inhibitory (IPSP). The output function is a static nonlinear function (sigmoid) that provides the average pulse density of action potentials fired by neurons depending on the sum of EPSPs and IPSPs at the input. This non-linear function accounts for threshold and saturation effects that take place at the somas and initial axonal segments of considered cells.

Formally, the input function is represented by a second order low-pass filter *H*(*s*) = *W*/(*s*+*1/*τ*_w_*)^2^ (where *s* is the Laplace variable). The impulse response of this filter is given by

(1) $$h(t)=\frac{W}{\tau_{w}}\cdot t\cdot e^{-t/\tau_{w}}$$

Parameters *W* and τ *_w_* are tuned such that *h*(*t*) approximates the shape of real excitatory (glutamatergic) or inhibitory (GABAergic) postsynaptic potentials (Lopes Da Silva et al., 1976). The quantity *W*.τ^2^*_w_* is the static gain of filter *h*. Lumped parameter τ*_w_* (expressed in s) is linked to the kinetics of synaptic currents. It determines both the rise time (*t_rise_* = τ*_w_*) and the decay time (*t_decay_* = 3.146τ*_w_*) of the second order filter impulse response *h* and it is usually adjusted with respect to the physiological rise and decay times of actual PSPs (Molaee-Ardekani et al., 2010). Given the time constantτ_*w*_, parameter *W* can be used to adjust the sensitivity of synapses (the maximal PSP amplitude is *W*.*e*^−1^). An alternative implementation of the *h* function was introduced in Bojak and Liley (2005) and is described in detail in Molaee-Ardekani et al. (2013). It is based on a bi-exponential pulse-to-wave function with two time constant parameters. This implementation allows for the separate adjustment of the rise and decay times of PSPs, and therefore a better approximation of actual PSPs in some circumstances. Besides, the output function is represented by $S(v)=\frac{2e_{0}}{1+e^{r(v_{0}-v)}}$, where 2*e*_0_ is the maximum firing rate, *v*_0_ is the postsynaptic potential corresponding to a firing rate of *e*_0_ and *r* is the steepness of the sigmoid.

#### The cortical compartment

The cortical compartment was inspired from an existing model of the neocortex which proved its capability of generating both normal and epileptiform activity. Readers may refer to Molaee-Ardekani et al. (2010) for details. In brief, the cortical compartment integrates a subpopulation of pyramidal cells (*P*, *W* = *A*_*C*_, τ*_w_* = τ*_ac_* in Equation 1) and two inhibitory neuronal populations (*I^c^*_1_ and *I^c^*_2_, Figure 1A) representing soma- and proximal-dendrite targeting interneurons (GABA*_A, fast_* currents, *W* = *G_C_*, τ*_w_* = τ*_gc_* in Equation 1) and dendrite-targeting interneurons (GABA*_A, slow_* currents, *W* = *B_C_*, τ*_w_* = τ*_bc_* in Equation 1), respectively. Pyramidal collateral excitation was implemented as in Jansen et al. (1993).

In addition, these three cortical subpopulations receive excitatory input from the thalamic compartment. Therefore, feed-forward inhibition (FFI) is represented in the model as the two subpopulations of interneurons project to the pyramidal subpopulation (see *The Thalamic and Reticular Compartments* paragraph below).

#### Short-term synaptic depression (STD)

STD is present in the neocortex (Boudreau and Ferster, 2005). It can be potentially involved in the context of direct stimulation of the thalamus as TC cells directly project to cortical pyramidal cells. Consequently, this mechanism was implemented at the interface of thalamic/cortical compartments. To our knowledge, an implementation of STD in neural mass models has not been proposed before.

In our model, we represented a modulatory effect of the amplitude of the average EPSP (parameter *A_C_*') at the level of subpopulation *P* depending on the density of action potentials [*d_AP_*(*t*)] coming from the thalamic compartment. This modulatory effect was obtained by multiplying *A_C_*' by a time-varying coefficient κ(*t*) ∈ [0.6, 1] where the function describing the evolution of κ(*t*) was derived from Chung et al. (2002). This study shows that: (i) cortical EPSPs drop by 40% under periodic low-frequency intense thalamocortical (*TC*) cell firing and, (ii) this drop in cortical EPSP is directly linked to transient depression of thalamocortical monosynaptic projections to pyramidal neurons.

In line with these observations, STD was implemented as follows. First, signal *d*^(*t*)^*_AP_* is low-pass filtered (cutoff frequency = 10 Hz) to restrict the STD effect to LFS. Then, from each time *t*_η_ at which the filtered signal *d^f^_AP_*(*t*) exceeds a firing rate equal to η, the κ(*t*) coefficient undertakes an exponential decay given by κ (*t*) = κ_η_ · *e*^−*t*/τ^ where κ_η_ = κ(*t*^−^_η_) and where *t*^−^_η_ is the time instant that just precedes *t*_η_. The decrease of κ(*t*) is limited to the time interval [*t*_η_ + 0.45 s] and cannot exceed 40%, total. Parameters η and τ were set to 0.8 and 8 s, respectively.

#### The thalamic and reticular compartments

The thalamic compartment was limited to one population of excitatory neurons (known as glutamatergic thalamocortical - *TC* - cells) receiving glutamatergic EPSPs (*W* = *A_Th_*, τ*_w_* = τ*_aTh_* in Equation 1) from cortical pyramidal cells (*P*) and GABAergic IPSPs with slow (*W* = *B_Th_*, τ*_w_* = τ*_bTh_* in Equation 1) and fast (*W* = *G_Th_*, τ*_w_* = τ*_gTh_* in Equation 1) kinetics from the reticular compartment (RtN). Here, we increased the time constant (τ*_bTh_*) with respect to τ*_bc_* to account for both GABA*_A, slow_*- and GABA*_B_*-receptor mediated currents in a single variable. *TC* cells directly target both cortical pyramidal cells and interneurons. The activation of these GABAergic interneurons subsequently promotes inhibition of pyramidal cells after a di-synaptic delay. Therefore, *TC* cells activation induces first an EPSP followed later on by an IPSP on cortical pyramidal cells, resulting in feed-forward inhibition (FFI). The *RtN* compartment comprised two inhibitory subpopulations, namely *I^RT^*_1_ and *I^RT^*_2_ which both receive excitatory input from the cortical (*W* = *A_Rt_*, τ*_w_* = τ*_aRt_* in Equation 1) and the thalamic (*W* = *A_Rt_*, τ*_w_* = τ*_aRt_* in Equation 1) compartments.

#### Simulation of stimulation effects

Stimulation currents induce a perturbation of the membrane potential of neurons. At cellular level, this effect can be accounted for by the “λ*E* model”, which is well-grounded in the biophysics of compartment models (Rattay, 1998; McIntyre et al., 2004a; Manola et al., 2005, 2007) (see Miranda et al., 2009 for a review) and supported by *in vitro* experiments (Bikson et al., 2004; Frohlich and McCormick, 2010). This model $\Delta V\approx \vec{\lambda }.\vec{E}$ approximates the membrane potential variation Δ*V* as a linear function of the electrical field $\vec{E}$ induced by stimulation ($\vec{\lambda }$ representing the membrane space constant). In our neural mass model, the situation is less straightforward as space is not explicitly represented, conversely to detailed or mean-field models. However, within a certain range of intensity values, it has been shown that the membrane potential variation Δ*V* is modified in a linear way with respect to the electrical field which is itself proportional to the stimulation intensity (Bikson et al., 2004). These considerations led us to also assume a linear variation for the mean membrane potential as a function of stimulation intensity, in stimulated sub-populations of neurons. In addition, stimulation was represented by a train of periodic monophasic depolarizing pulses. The pulse width was fixed to 1 ms (as in clinics). Pulses were low-pass filtered to account for the average time of repolarization (set to 4.8 ms) in stimulated sub-populations of cells. The resulting stimulation signal was added to the mean membrane potential of neuronal sub-populations included in the thalamic (*TC*) and reticular (*I^RT^*_1_ and *I^RT^*_2_) compartments of the proposed model. The depolarizing effect was weighted by three coefficients *S_TC_*, *S*_*Rt*1_ and *S*_*Rt*2_ (Table 1) accounting for the possibly different stimulation impact at the thalamic and reticular level.

**Table 1:** **Model parameters, values and interpretation**.

**Parameter**	**Value**	**Interpretation**
*A_C_*	6 (optimized, pathological) 3 (normal) mV	Amplitude of the cortical average EPSP
*A_C_*'	κ*(t).A_C_* mV	Amplitude of the cortical average EPSP in response to thalamic input (only on subpopulation *P*)
*B_C_*	14 (optimized, pathological) 50 (normal) mV	Amplitude of the cortical average IPSP (GABA*_A, slow_* mediated currents)
*G_C_*	16.5 (optimized, pathological) 22 (normal) mV	Amplitude of the cortical average IPSP (GABA*_A, fast_* mediated currents)
*A_Th_*	3.5 mV	Amplitude of the thalamic average EPSP
*B_Th_*	30 mV	Amplitude of the thalamic average IPSP (GABA*_A, slow_* and GABA*_B_* receptors)
*G_Th_*	22 mV	Amplitude of the thalamic average IPSP (GABA*_A, fast_* receptors)
*A_Rt_*	3.5 mV	Amplitude of the reticular average EPSP
τ*_ac_*	1/80 s	Time constant of cortical glutamate-mediated synaptic transmission.
τ*_bc_*	1/35 s	Time constant of cortical GABA-mediated synaptic transmission (GABA*_A, slow_* receptors)
τ*_gc_*	1/180 s	Time constant of cortical GABA-mediated synaptic transmission (GABA*_A, fast_* receptors)
τ*_aTh_*	1/100 s	Time constant of thalamic glutamate-mediated synaptic transmission
τ*_bTh_*	1/20 s	Time constant of thalamic GABA-mediated synaptic transmission (GABA*_A, slow_* and GABA*_B_* receptors)
τ*_gTh_*	1/150 s	Time constant of thalamic GABA-mediated synaptic transmission (GABA*_A, fast_* receptors)
τ*_aRt_*	1/100s	Time constant of reticular glutamate-mediated synaptic transmission
ν_0_, *e*_0_, *r*	ν_0_ = 6 mV, *e*_0_ = 2.5 s^−1^ *r* = 0.56 mV^−1^	Parameters of the nonlinear sigmoid function (transforming the average membrane potential to an average density of action potentials)
*C*_*P*−*P*'_	135	Collateral excitation connectivity constant
*C*_*P*'−*P*_	108	Collateral excitation connectivity constant
*C*_*P*−*I*^*C*^_2__	33.75	*P* to *I*^*C*^_2_ connectivity constant
*C*_*I*^*C*^_2_−*P*_	33.75	*I*^*C*^_2_ to *P* connectivity constant
*C*_*P*−*I*^*C*^_1__	40.5	*P* to *I*^*C*^_1_ connectivity constant
*C*_*I*^*C*^_2_−*I*^*C*^_1__	13.5	*I*^*C*^_1_ to *I*^*C*^_2_ connectivity constant
*C*_*I*^*C*^_1_−*P*_	91.125	*I*^*C*^_1_ to *P* connectivity constant
*C*_*TC*−*P*_	120	*TC* to *P* connectivity constant
*C*_*TC*−*I*^*C*^_1__	30	*TC* to *I*^*C*^_1_ connectivity constant
*C*_*TC*−*I*^*C*^_2__	45	*TC* to *I*^*C*^_2_ connectivity constant
*C*_*TC*−*I*^*Rt*^_1__	20	*TC* to *I*^*RT*^_1_ connectivity constant
*C*_*TC*−*I*^*Rt*^_2__	20	*TC* to *I*^*Rt*^_2_ connectivity constant
*C*_*P*−*I*^*Rt*^_1__	30	*P* to *I*^*RT*^_1_ connectivity constant
*C*_*P*−*I*^*Rt*^_2__	30	*P* to *I*^*Rt*^_2_ connectivity constant
*C*_*P*−*TC*_	20	*P* to *TC* connectivity constant
*C*_*I*^*Rt*^_1_−*TC*_	35	*I*^*Rt*^_1_ to *TC* connectivity constant
*C*_*I*^*Rt*^_2_−*TC*_	5	*I*^*Rt*^_2_ to *TC* connectivity constant
μ_*P*1_	0	Mean of nonspecific cortical input
μ_*P*2_	70	Mean of nonspecific subcortical input
σ_*P*1_	20.v6	Standard deviation of nonspecific cortical input
σ_*P*2_	35.v6	Standard deviation of nonspecific subcortical input
*S*_*TC*_	5	Stimulation impact on subpopulation *TC*
*S*_*Rt*1_	4	Stimulation impact on subpopulation *I*^*Rt*^_1_
*S*_*Rt*2_	4	Stimulation impact on subpopulation *I*^*Rt*^_2_
*f*_*s*_	1 – 150Hz	Frequency of the stimulation signal (pulse train)
*A*_*fs*_	1	Stimulation signal amplitude

*Model parameters used to reproduce LFPs_FCD_. Stimulation impact parameters S_TC_, S_Rt1_ and S_Rt2_ are set to zero during the simulation of the NS scenario. These parameters are held constant for all other stimulation scenarios*.

#### Model parameters, outputs, and implementation

Parameter values as well as physiological interpretation are provided in Table 1. Note that each synaptic connection in the model is weighted by a connectivity constant denoted by *C*_*SP*1−*SP*2_ where *SP1* and *SP2*, respectively, denote the source and target subpopulations. In addition, two Gaussian noise inputs *p*_*P*_(*t*)~ *N*(μ_*P*_, σ_*P*_) and *p*_*TC*_(*t*)~ *N*(μ_*TC*_, σ_*TC*_) were used to represent nonspecific inputs on pyramidal and thalamocortical cell subpopulations. Finally, signals simulated at the level of pyramidal cells in the cortical compartment and at the level of *TC* cells in the thalamic compartment were chosen as model outputs. They correspond to the sum of PSPs at each compartment respectively. The temporal dynamics of these signals provide a good approximation of actual LFPs. The model was implemented in Simulink®, and all other complementary scripts were implemented in MATLAB®.

### Real data for model tuning

We used real clinical data to tune the model into a functioning mode which simulates pathological activity. The clinical data set was limited to a unique patient who underwent thalamic DBS during the presurgical intracerebral EEG exploration (iEEG performed with depth electrodes implanted under stereotaxic conditions) at the Epilepsy Surgery Unit, Rennes University Hospital. This particular patient was chosen for two main reasons: (1) the pronounced frequency-dependent stimulation effects observed during his preoperative diagnostic iEEG exploration at LF, IF and HF in addition to (2) the existence of an epileptogenic zone in a limited area of the premotor cortex (PMC).

**Figure 2:**
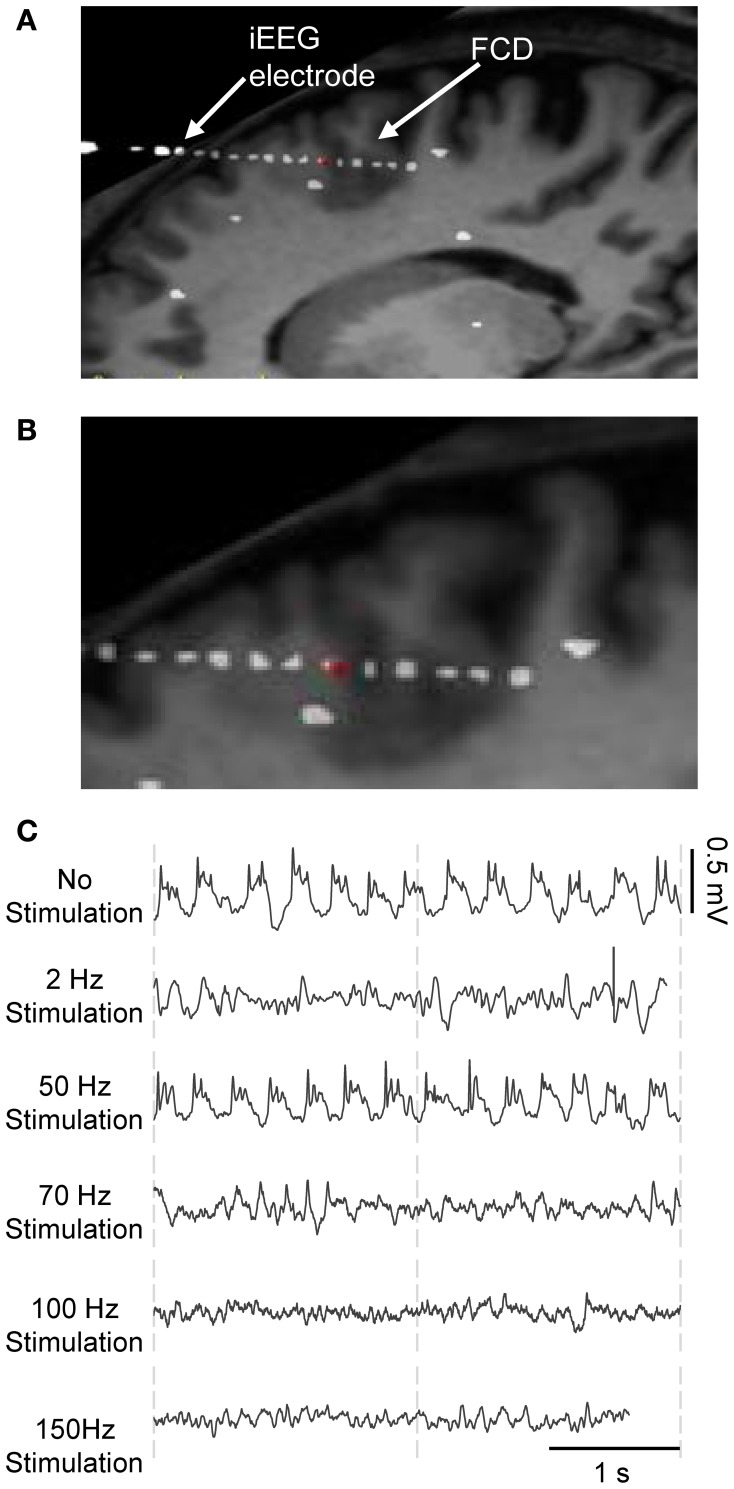
**Frequency-dependent stimulation effects: real data.** iEEG signals recorded during presurgical depth-EEG exploration in a patient with drug-resistant epilepsy. **(A)** MRI data showing the FCD (focal cortical dysplasia in the PMC) and the electrode trajectory. The red dot marks the position of the depth electrode in the FCD. **(B)** Zoom on the FCD. **(C)** DBS of the CMN modulated the pathological activity of the FCD in a frequency-dependent manner. LFS (2 Hz) and HFS (≥70 Hz) suppressed pathological oscillations. IFS (50 Hz) had no effects.

In brief, this patient suffered from partial drug-resistant epilepsy since the age of two. MRI scans and EEG recordings pointed out the existence of a neuronal malformation known as FCD in the PMC at the origin of seizures. This type of cortical malformation is known for its epileptogenic features like neuronal hyperexcitability and hypersynchronization and its characteristic epileptiform discharges (continuous, rhythmic or semirhythmic spikes, and polyspikes) (Avoli et al., 2003; Palmini, 2010) as shown in Figure 2C. Based on various clinical studies reporting the modulation of epileptic cortical activity by the stimulation of the CM nucleus (Velasco et al., 1995, 1997, 2000, 2001, 2007), it was decided by neurologists and neursosurgeons to implant a depth electrode in this nucleus, as potentially beneficial for the patient who gave his informed consent.

During the presurgical exploration, the stimulation of the thalamic CM nucleus (CMN) induced frequency-dependent modulation of the pathologic activity of the FCD (Figure 2). Readers may refer to (Pasnicu et al., 2013) for detailed information. Interestingly, LFS (2 Hz, 4 mA) and HFS (70, 100, and 150 Hz, 0.8 mA) desynchronized the pathological activity of the FCD, while IFS (50 Hz, 0.8 mA) barely affected it. These segments of signals corresponding to either typical pathological activity or modulated activity (depending on stimulation conditions) were used to optimize the model parameters.

### Processing of real and simulated signals

**Figure 3:**
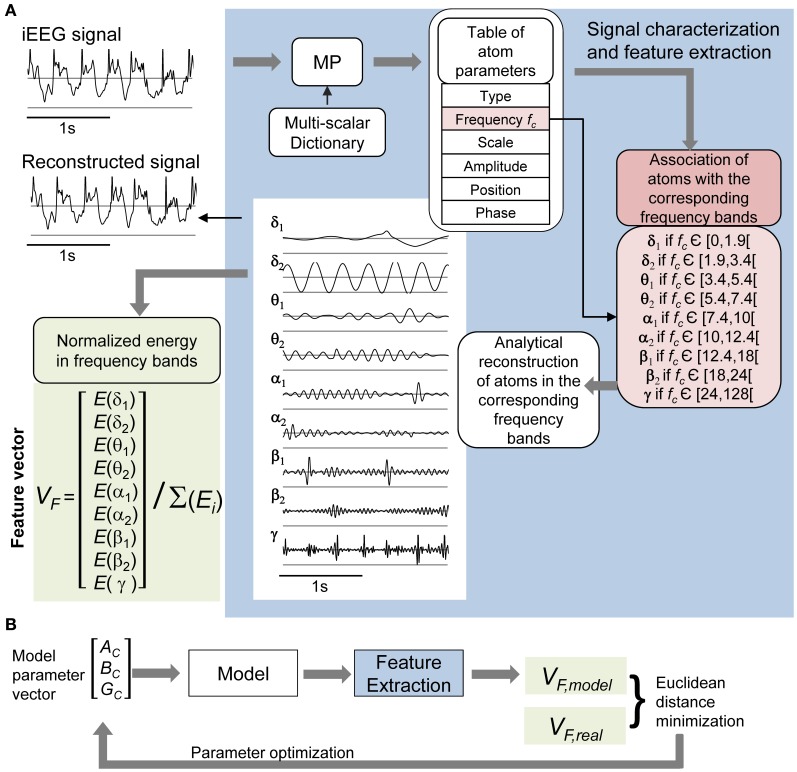
**iEEG signal processing. (A)** *Feature vector extraction*. Input signals were characterized using the matching pursuit (MP) method (dictionary of Gabor, Fourier, and Dirac atoms). Parameters of detected atoms (atom type, central frequency *f_c_*, scale, phase, amplitude, and position) are extracted by MP from input signals. Detected atoms are then associated with frequency bands (δ_1_ to γ) depending on their proper central frequency. Sub-band (δ_1_ to γ) signals were reconstructed from the sum of corresponding atoms, themselves obtained by fitting parameters into their analytic expression (see top left: input and reconstructed signals). The normalized energy vector [*E*(δ_1_) … *E*(γ)]/(*E*(δ_1_) + … + *E*(γ)] was chosen as the feature vector for further *optimization of model parameters*. **(B)** The model's free parameters *A_C_*, *B_C_*, and *G_C_* were optimized by minimizing the distance between the feature vector *V_F, model_* of the simulated cortical LFP and the average of real feature vectors *V_F, real_* of LFPs_FCD_.

The use of signal processing techniques was necessary (i) to quantify the above-described effects of stimulation in real iEEG signals, and (ii) to define a feature-vector-based cost function for model parameter optimization. Figure 3A illustrates the feature extraction methodology. iEEG signals recorded in the FCD in absence of stimulation (LFPs_FCD_) and under different stimulation conditions were decomposed using an orthogonal matching pursuit algorithm [matching pursuit toolkit—MPTK—(Krstulovic and Gribonval, 2006)]. First introduced in 1993 (Mallat and Zhifeng, 1993), matching pursuit is signal processing algorithm used to decompose any time series into a linear sum of waveforms selected from a predefined dictionary based on a mother wavelet. To proceed, a proper multi-scalar dictionary of Gabor, Fourier, and Dirac atoms was first defined to account for real iEEG signal components (time-frequency atoms are waveforms well localized in both the time and the frequency domains). In line with (Krstulovic and Gribonval, 2006), the multi-scalar dictionary was formed by translation in time and amplitude/frequency modulation of atoms (defined as Gabor and Fourier functions in our case), over ten different user-defined time scales (i.e., the atom durations, ranging from 0.125 to 5 s). Then, the algorithm provided a table of time-frequency parameters associated to the detected atoms (atom type, central frequency, phase, scale, amplitude, position). Identified atoms were reconstructed using the extracted parameter table and their analytical expression. They were then associated to a given frequency band depending on their central frequency. These frequency bands corresponded to the classical EEG bands as defined in normal adults (δ_1_ [0–1.9Hz], δ_2_ [1.9–3.4 Hz], θ_1_ [3.4–5.4 Hz], θ_2_ [5.4–7.4 Hz], α_1_ [7.4–10 Hz], α_2_ [10–12 Hz], β_1_ [12–18 Hz], β_2_ [18–24 Hz], γ [24–128 Hz]) (Figure 3A, blue). Finally, a 9D feature vector *V_F_* was defined from the normalized energy distribution in these frequency bands, itself computed as the sum of averaged (over time) atom energies relative to the total signal energy (Figure 3A, green).

### Model optimization under the “no stimulation” condition

In order to simulate LFPs_FCD_, we optimized the excitation/inhibition ratio of the cortical compartment. Thus, the average EPSP/IPSP amplitude parameters of the cortical compartment {*A_C_, B_C_, G_C_*} were considered as free parameters while all other model parameters were set to fixed values (Table 1). The optimization method is illustrated in Figure 3B. For each triplet {*A_C_, B_C_, G_C_*}, the feature vector *V_F, model_* of the model's output signal (cortical compartment's LFP) was calculated and compared to *V_F, real_*, i.e., the feature vector computed from the average of the 20 feature vectors, each computed on a 5 s signal segment of real LFPs_FCD_. Feature vectors *V_F, model_* and *V_F, real_* were computed as described in section Processing of Real and Simulated Signals. The optimization procedure aimed at finding the triplet $\left\{\overset{⌢}{{\text{A}}_{\text{C}}},\;\overset{⌢}{{\text{B}}_{\text{C}}},\;\overset{⌢}{{\text{G}}_{\text{C}}}\right\}$ that minimizes a cost function simply corresponding to the Euclidean distance *d*(*V_F, real_*, *V_F, model_*) when parameters *A_C_*, *B_C_*, and *G_C_* span predefined ranges of values according to a Brute-Force procedure.

## Results

In this section, results regarding the identification of cellular mechanisms underlying the modulation of cortical activity by thalamic DBS are reported. First, the model capability to reproduce signals similar to those recorded from the FCD in the patient was assessed, under two conditions (no stimulation and during stimulation). Three mechanisms contributing to frequency-dependant stimulation effects could be identified. Then, simulations were performed to analyze the marginal or joint contribution of these mechanisms at low, intermediate or high frequency stimulation.

### Simulation of LFPs_FCD_ under no stimulation condition

**Figure 4:**
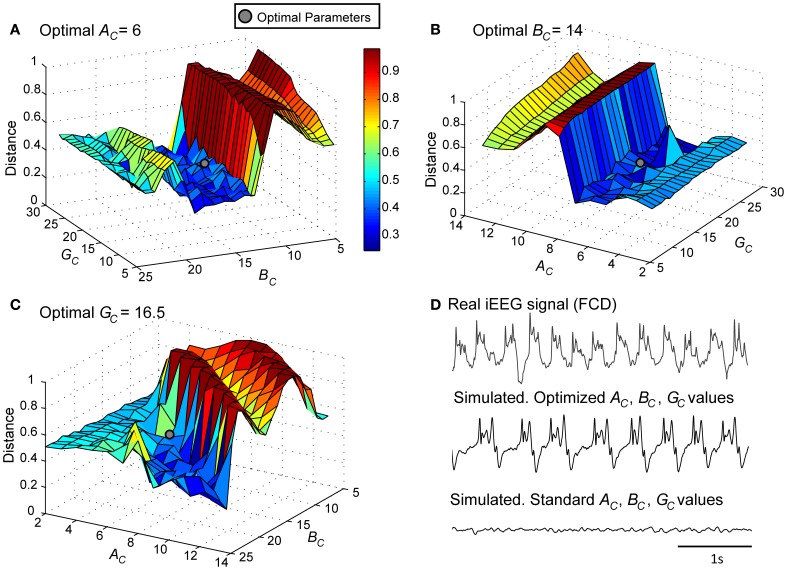
**Model parameter optimization.** Normalized Euclidian distance between *V_E, real_* and *V_E, model_*. Best fit (gray disk) between simulated and real LFPs_FCD_ was obtained for **(A)** *A_C_* = 6, **(B)** *B_C_* = 14, and **(C)** *G_C_* = 16.5. **(D)** For these modified values of excitation and inhibition, the simulated signal exhibits similar characteristics as the iEEG signal recorded in the FCD. For standard values of excitation and inhibition (*A_C_* = 3, *B_C_* = 50, *G_C_* = 22), the model generates background EEG activity.

As a first step, we verified the ability of the model to generate signals that resemble those recorded from the FCD in the considered patient (*LFPs*_FCD_). This procedure, described in sections Processing of Real and Simulated Signals and Model Optimization Under the “No Stimulation” Condition, led us to identify a minimal distance (Figures 4A–C) and thus an optimal parameter vector $\left\{\overset{⌢}{{\text{A}}_{\text{C}}},\;\overset{⌢}{{\text{B}}_{\text{C}}},\;\overset{⌢}{{\text{G}}_{\text{C}}}\right\}=\{6,14,16.5\}$ for which simulated signals under the no stimulation condition have similar features as compared with those of real signals (Figure 4D).

### Simulation of LFPs_FCD_ under stimulation conditions

**Figure 5:**
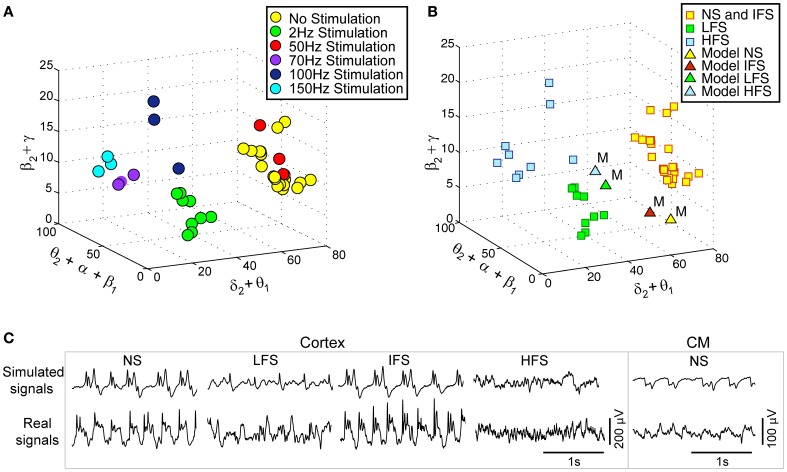
**Characterization and classification of real and simulated data. (A)** 3-dimensional (3D) projection of feature vectors (*V_F, real_*) corresponding to different stimulation conditions. This projection was obtained by summing some coordinates of initial 9D feature vectors to get 3D vectors [*E*(δ_2_)+*E*(θ_1_), *E*(θ_2_)+*E*(α_1_)+*E*(α_2_)+*E*(β_1_), *E*(β_2_)+*E*(γ)]/[*E*(δ_1_)+… +*E*(γ)]. Each vector was then represented by a point in the 3D space (δ_2_+θ_1_, θ_2_+α_1_+α_2_+β_1_, β_2_+ γ).Three main classes can be visually identified. **(B)** Clusters obtained using the k-means algorithm (Mahalanobis distance). The three clusters correspond to (i) low-frequency stimulation (LFS) effects (green squares), (ii) no stimulation (NS) and intermediate-frequency stimulation (IFS) effects (yellow squares), and (iii) high-frequency stimulation (HFS) effects (blue squares). Simulated signals corresponding to the four types of scenarios (NS, LFS, IFS, and HFS) were also projected in the same space (triangles). **(C)** Two-second segments of real and simulated signal during NS, LFS, IFS, and HFS.

Actual LFPs_FCD_ recorded at various stimulation frequencies (2, 50, 70, 100, and 150 Hz) were first characterized using the matching pursuit method described in section Processing of Real and Simulated Signals (Figure 3A). Results are shown in Figure 5A where feature vectors of segments of LFPs_FCD_ are represented in a 3D space where axes correspond to merged typical EEG frequency bands (δ_2_ to θ_1_, θ_2_ to β_1_, β_2_ to γ). Results show that the distribution of points in the 3D frequency space is not random but clustered, indicating that the frequency content of LFPs_FCD_ segments depends on the stimulation frequency. In addition, some clusters are very close. This is typically the case for i) the no stimulation (yellow) and the 50 Hz stimulation conditions (red) on the one hand, and ii) the 70 Hz (violet) and 150 Hz (cyan) stimulation conditions on the other hand. To go beyond the qualitative clustering performed by visual inspection of 3D plots, a K-means clustering algorithm implemented in MATLAB and using a Mahalanobis distance was used to automatically detect the three types of stimulation effects. Initial centroids were randomly chosen. The optimal clustering that globally minimizes intra-cluster inertia is presented in Figure 5B. LFPs_FCD_ segments were automatically classified into three subgroups. The first subgroup contains LFPs_FCD_ segments obtained for low-frequency stimulation (LFS). The second subgroup gathers all segments recorded for high frequency stimulations (HFS, > 70 Hz). And finally, in the third subgroup, segments obtained under the no stimulation and the intermediate stimulation frequency (IFS, 50 Hz) conditions are merged together, suggesting that this stimulation frequency does not reduce the “epileptiform aspect” of the activity reflected in the LFP.

**Figure 6:**
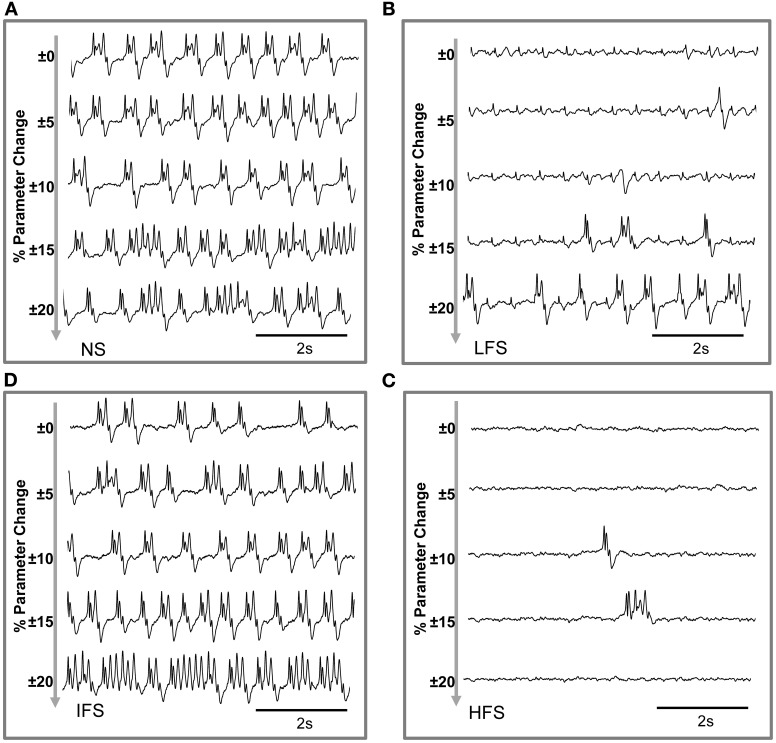
**Evaluation of parameter sensitivity.** Model output sensitivity to variations of excitatory and inhibitory key parameters. Realizations of parameter vector Θ = {*A_C_, B_C_, G_C_, A_Th_, B_Th_, G_Th_, A_Rt_*} were randomly (uniform law) generated around the optimal parameter vector Θ_0_ over a variation domain defined by (1 ± ζ) · Θ_0_. For ζ ≤ 0.2 (±20% variation), stimulation effects are preserved in the model for **(A)** no stimulation, **(B)** low-frequency stimulation, **(C)** intermediate-frequency stimulation, and **(D)** high-frequency stimulation.

Based on this characterization of local field potentials recorded in the FCD (LFPs_FCD_), parameters *S_TC_*, *S*_*Rt*1_ and *S*_*Rt*2_ were manually tuned to lead the model to generate simulated signals which have spectral characteristics similar to those of actual LFPs_FCD_. Such a manual procedure was sufficient to reproduce stimulation effects observed in one patient. However, extending the study to a larger group of patients would have made imperative an automated parameter fitting procedure based on the spectral characteristics of real EEG signals as in Rowe et al. (2004). Figure 5B shows the projection of representative simulated LFPs_FCD_ in the 3D frequency space (“M” triangles). As depicted, simulated signals obtained for LFS, IFS and HFS were close to corresponding clusters obtained from real signals for the exact same computation of feature vectors. Shown in Figure 5C, these representatives simulated LFPs_FCD_ do not perfectly match actual signals. However, qualitatively similar bifurcations were observed in the model when the stimulation conditions are changed. Indeed, under the no stimulation (NS) and the IFS condition the model generates rhythmic slow oscillations (δ) with superimposed faster activity (β, γ), as observed in real data. For LFS and HFS conditions, strong modulation of this activity was also obtained in the model. At LFS, in the model, the slow wave activity was strongly reduced but spike events occurred in the signals at the instant times of stimulation, mimicking, to some extent, comparable events also present in actual LFPs_FCD_. Finally, at HFS, slow oscillations (δ) were abolished in the model which generates quasi-normal background activity. This simulated activity was also comparable to real activity observed for HFS stimulation but disclosed less γ activity. Note that these are the effects which were quantified in Figure 5B. The qualitative optimization procedure of parameters *S_TC_*, *S*_*Rt*1_, and *S*_*Rt*2_ was then complemented by an evaluation of parameter sensitivity aimed at studying the impact of random changes affecting the parameter vector Θ = {*A_C_, B_C_, G_C_, A_Th_, B_Th_, G_Th_, A_Rt_*} on simulated signals. Parameter vector Θ determines the excitability properties in the three model compartments. As shown in Figure 6, results show that the simulated signals obtained under the four stimulation conditions (NS, LFS, IFS, HFS) stay “quite robust” (in the sense that waveforms are conserved) when parameters stay in the range [$\left[\overset{⌢}{\Theta }\pm \zeta \cdot \overset{⌢}{\Theta }\right]$] with 0 ≤ ζ ≤ 0.2.

### Mechanisms underlying frequency-dependant stimulation effects

Three main mechanisms implemented in the model are required to mimic actually observed effects of the CM nucleus stimulation. These mechanisms are the following: (i) the presence of feed-forward inhibition (FFI) at the level of thalamic projections to the FCD, (ii) the presence of short-term depression (STD) at the level of the thalamocortical glutamatergic synapses and, (iii) the depolarization of RtN inhibitory interneurons targeting *TC* cells.

**Figure 7:**
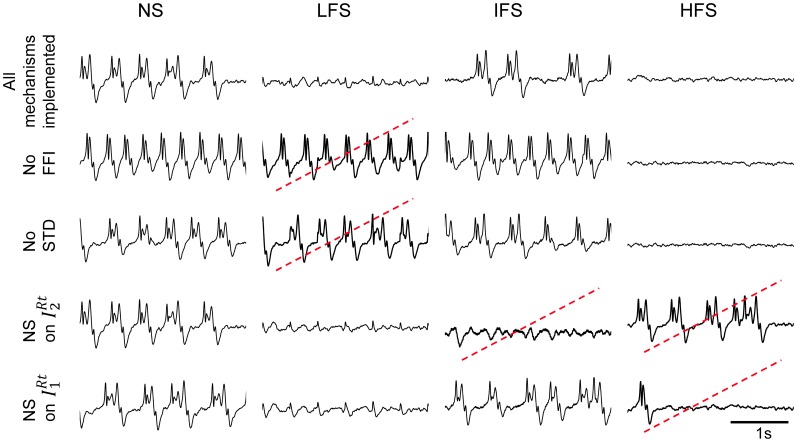
**Conditions to reproduce frequency-dependent stimulation effects.** Model output in the case where one of the implemented mechanisms (FFI, STD, depolarization of *I*^*Rt*^_2_, and *I*^*Rt*^_1_ „ respectively) is removed at a time. LFS effects are not reproduced when the model does not account for FFI and STD. HFS effects require the depolarization of both reticular populations *I*^*Rt*^_2_ and *I*^*Rt*^_1_. Suppression of epileptic activity is observed at IFS when *I*^*Rt*^_2_ interneurons are removed. Red dotted lines indicate situations where simulated signals do not match real ones for given stimulation condition.

This result raises an additional question: to what extent the joint effect of these mechanisms is necessary to reproduce frequency-dependant stimulation effects (LFS, IFS, and HFS). In order to assess their individual contribution, we performed simulations where each mechanism was either present in—or removed from—the model (the model parameters remaining unchanged). Results are displayed in Figure 7. First, they confirmed that both FFI and STD mechanisms are jointly necessary in the model to suppress the epileptic activity in the FCD when LFS is being used since the withdrawal of either STD or FFI leads the model to generate epileptic activity at LFS. Second, results indicated that the RtN inhibitory interneurons targeting *TC* cells (both *I*^*RT*^_1_ and *I^RT^*_2_ subpopulations) must be affected (i.e., depolarized) by the stimulation to obtain a suppression of epileptic activity when HFS is being used, as observed in the patient. Third, and interestingly, an unexpected effect was observed at IFS when the depolarization of *I^RT^*_2_ interneurons was removed from the model. Indeed, epileptic activity was abolished in this case, which is really unlikely to occur during actual stimulation as both subtypes of neurons are expected to be affected by the direct stimulation of the CM nucleus.

**Figure 8:**
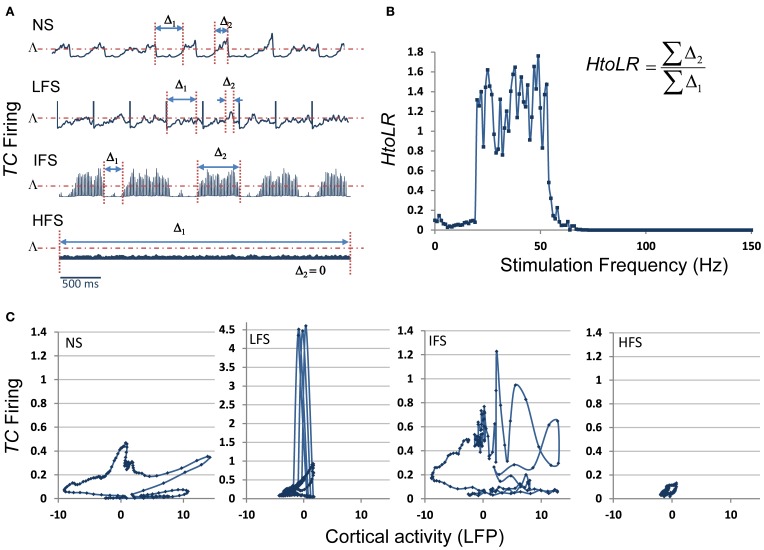
**Model behavior as a function of the stimulation frequency. (A)** The firing rate of TC cells depends on the stimulation frequency (Δ_1_: time interval for which this firing is lower than a threshold Λ, Δ_2_: time interval for which this firing is higher than Λ). **(B)** Evolution of the “High to Low firing Ratio” (*HtoLR*) as a function of stimulation frequency. **(C)** Phase portraits (FCD activity vs. CM firing) for the four stimulation conditions (NS, LFS, IFS, and HFS).

These results were complemented by a deeper analysis of the thalamic output (i.e., the firing rate of *TC* cells) in response to stimulation at low, intermediate and high frequency. Results are provided in Figure 8. First, they showed that the thalamic output dramatically differs depending on the stimulation frequency (Figure 8A). Under the no stimulation condition, the firing rate continuously oscillates around a certain value (referred to as Λ, Figure 8A). At LFS, the firing rate was found to be lower, except at the stimulation times where it abruptly and transiently increased. At IFS, a balance was observed between time intervals for which the *TC* firing is above and below Λ. Finally, at HFS, the output of *TC* cells was found to be very low, i.e., systematically under the threshold Λ. From these observations, we could define (i) two time intervals, Δ1 and Δ2, for which the *TC* cells firing rate is either below Λ(Δ1) or above Λ (Δ2) and (ii) a “high to low firing” ratio (*HtoLR*) which provides an indication on the amount of time the *TC* cells spend firing (up state) relatively to the amount of time they do not fire (down state). Figure 8B provides the evolution of the *HtoLR* when the stimulation frequency is progressively changing from 0 to 150 Hz in the model. As depicted, these simulations indicated that three stimulation frequency ranges have dramatic effects on the firing of *TC* cells. First, from 0 to 20 Hz, the down state is predominant. Then, an abrupt jump was observed around 22 Hz indicating that beyond this value, the firing rate dramatically increased. Interestingly, from 55 to 65 Hz, a progressive decrease of the *HtoLR* was observed. Then, after 70 Hz, the ratio is equal to zero indicating that *TC* cells did not fire anymore. Finally, in order to relate the thalamic activity with the cortical activity, we plotted the phase portraits (*TC* cell firing vs. cortical LFP) as illustrated in Figure 8C. Results confirmed the visual inspection of signals simulated at the two sites. For the no stimulation (NS) and for the intermediate frequency stimulation (IFS) conditions, phase portraits were found to be quite similar. They indicated the presence of mixed slow/fast oscillations in both signals. For the low frequency stimulation (LFS) condition, oscillations in the simulated LFP in the FCD were reduced. They came along with short-duration, abrupt and rhythmic augmentations of the *TC* firing corresponding to stimulation pulses. Finally, for the high frequency stimulation (HFS) condition, oscillations in both types of activity stayed confined to small amplitude values.

## Discussion

We modeled the thalamocortical loop in order to investigate frequency-dependent effects of electrical stimulation performed in the thalamus and aimed at modulating the neocortical activity. We chose to elaborate our model at a mesoscopic level, i.e., intermediate between microsocopic and macroscopic.

Regarding the model architecture, we followed a similar approach to that used in previously proposed models of the thalamocortical loop (Robinson et al., 2002; Suffczynski et al., 2004; Breakspear et al., 2006; Roberts and Robinson, 2008; Marten et al., 2009; Crunelli et al., 2011). Our model includes three main compartments: cerebral cortex, reticular nucleus and thalamic relay. Subpopulations of neurons and interneurons located in these three structures interact via excitatory and/or inhibitory synaptic connections. The novelty with respect to aforementioned studies is threefold. First, we modified the cortical compartment in order to better approximate the temporal dynamics of epileptic signals recorded in the FCD. This modification consisted in the use of two types of interneurons (mediating GABAergic IPSPs with slow and fast kinetics on cortical principal cells), as reported in a previous study (Molaee-Ardekani et al., 2010). Second, our model accounts for the direct effects of electrical stimulation. At this stage, we used the $\Delta V\approx \vec{\lambda }\cdot \vec{E}$ assumption according to which the perturbation of the mean membrane potential of neurons is a linear function of the electrical field magnitude induced by bipolar stimulation. This “λE” assumption was already used in neural mass models in the context of low-intensity direct hippocampal stimulation to anticipate seizures (Suffczynski et al., 2008) as well as in the analysis of the stimulus-response relationship of DBS in healthy animals (Adhikari et al., 2009). However, it is worth mentioning that in our model, the three subtypes of neurons (*TC* cells and both subpopulations of inhibitory neurons in the RtN) are depolarized by the stimulation, as suggested in (Molaee-Ardekani et al., 2013) and conversely to (Adhikari et al., 2009) where only principal cells are impacted. And third, our model includes two well-known mechanisms at the cortical level: feed-forward inhibition (FFI) and short-term depression (STD).

As in any modeling approach, our approach has some limitations. First, the chosen modeling level does not allow for analyzing sub-cellular mechanisms involved in stimulation-evoked changes. Similarly, it does not account for direct activation of axons by stimulation vs. somatic inhibition (McIntyre et al., 2004b), nor for the mechanisms of orthodromic/antidromic propagation of action potentials due to stimulation (Degos et al., 2005; Hammond et al., 2007; Dorval et al., 2008). Second, a strong assumption in the type of model we used (neural mass) is related to the intrinsic synchronization among neurons included in a given sub-population. This assumption does not allow for representing either de- or weakly-synchronized firing patterns that may be observed during epileptic activity, in particular during high frequency oscillations that can be encountered in FCDs (Brázdil et al., 2010). Nevertheless, we could accurately reproduce the abnormal rhythms generated in the FCD suggesting that main pyramidal cells have a relatively synchronized activity in this epileptogenic tissue. Third, regarding plasticity-related mechanisms, we only implemented short-term effects (i.e., STD) and neglected long-term plastic changes that may be induced by DBS (Shukla et al., 2013).

**Figure 9:**
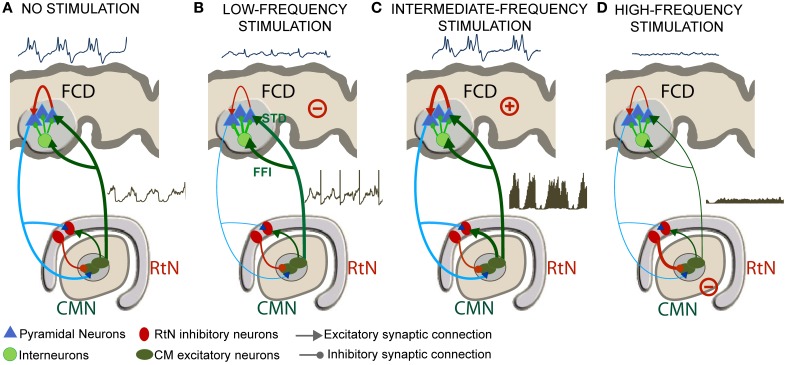
**Frequency-dependent mechanisms underlying DBS. (A)** Under the no stimulation (NS) condition, the thalamocortical loop is responsible for pathological oscillatory rhythms observed in the FCD. **(B)** For low-frequency stimulation (LFS), feed-forward inhibition (FFI, i.e., excitation of inhibitory cortical interneurons by *TC* cells) and short-term depression (STD, i.e., decreased excitatory synaptic efficacy in thalamus-to-cortex connections) was found to play a major role for the abortion of epileptic activity in the FCD. **(C)** For the intermediate-frequency stimulation (IFS) condition, thalamic output is reinforced (increase of *TC* cells firing) leading to an increase of the average excitatory postsynaptic potential (EPSP) on cortical pyramidal cells and to no “anti-epileptic” effect. **(D)** For high-frequency stimulation (HFS), the direct and sustained excitation of reticular nucleus (RtN) interneurons leads to dramatic decrease of *TC* cells firing rate and to a suppression of epileptic activity.

Despite these limitations, we could identify a number of mesoscopic factors which could explain the frequency-dependent mechanisms of thalamic stimulation. The model was tuned using electrophysiological data recorded in a patient in whom the centromedian nucleus (CMN) stimulation was particularly efficient to reduce the epileptic activity of a FCD located in the premotor cortex, in a frequency-specific manner. The main findings are summarized in Figure 9.

### “No stimulation” (NS) condition

In the model, under the NS condition, excitation among pyramidal cells had to be increased and inhibition had to be reduced in the cortical compartment for producing “pathological” oscillatory rhythms, as observed in the FCD. The thalamocortical loop was found to be responsible for these pathological dynamics, characteristic of FCDs. These findings are in line with histological studies showing that these typical oscillations are generated in altered brain tissue, where inhibition is partially deteriorated or dysfunctioning (Calcagnotto et al., 2005), and where excitation is heavily increased (Avoli et al., 2003). In addition to neuron alterations in the dysplastic tissue (Sisodiya et al., 2009), FCD keeps sufficient projections to—and input from—other brain structures to propagate pathological dynamics (Avoli et al., 2003). As mentioned, the presence of connections with subcortical structures was a necessary condition in the model for producing pathological oscillations resembling those actually recorded in the FCD (Figure 9A).

### Low-frequency stimulation (LFS) condition

For the low-frequency stimulation (LFS, *f* < 20 Hz) condition, two mechanisms were found to play a major role for the abortion of epileptic activity in the FCD: short-term depression (STD, i.e., decreased excitatory synaptic efficacy in thalamus-to-cortex connections) and feed-forward inhibition (FFI, i.e., excitation of inhibitory cortical interneurons by *TC* cells) (Figure 9B).

STD was reported in previous studies concerning cortical adaptation to thalamic stimulation, and suggesting that electrical LFS of *TC* cell axons *in vivo* resulted in a 40% reduction in cortical EPSPs (Chung et al., 2002). In the same context, LFS trains in adult anaesthetized rats provoked transient long-term depression of thalamocortical synapses; this was measured by up to 40% drop in cortical EPSPs after LFS trains and under the effect of GABA antagonist (Speechley et al., 2007).

As mentioned above, the LFS effects could not be reproduced by the model without incorporating also FFI. Actually, thalamocortical ascending fibers directly target pyramidal neurons as well as cortical GABAergic interneurons inducing EPSPs in both cell types (Pouille and Scanziani, 2001). In the model, while less efficient (STD) thalamic EPSPs arrive directly onto pyramidal neurons, IPSPs induced by thalamic stimulation also arrive on pyramidal neurons (FFI) lagging by 1–2 ms. This short latency between the onset of thalamocortical excitation and the onset of feed-forward inhibition presents a temporal “window of opportunity” for pyramidal cells to integrate excitatory and inhibitory inputs, thus keeping the transmembrane potential below firing threshold. In the literature, neuroanatomical and neurophysiological studies (Isaacson and Scanziani, 2011) showed the functional importance of FFI in regulating cortical dynamics by controlling cortical excitability (Gabernet et al., 2005). Our study suggests that LFS regulates cortical excitability by a dual mechanism of FFI and STD (Figure 9B).

### Intermediate-frequency stimulation (IFS) condition

For the intermediate-frequency stimulation (IFS, 20 < *f* < 70 Hz) condition, results indicated that the thalamic output is reinforced (increase of *TC* cells firing) and leads to an increase of the average excitatory postsynaptic potential (EPSP) on cortical pyramidal cells (Figure 9C). This effect corresponds to an increase of the spatiotemporal summation of unitary EPSPs. In this case, both the cortical excitability and the gain in the excitatory thalamocortical loop is increased, leading to “no anti-epileptic” effect. We did not find much studies using DBS stimulation in the intermediate frequency range of (20–60 Hz) in the context of epilepsy. Nevertheless, it is noteworthy that 50 Hz stimulation frequency is classically used during the presurgical evaluation of patient with intractable partial epilepsy in order to trigger seizures and delineate the epileptogenic zone (Talairach et al., 1974; Jayakar et al., 1992). The same frequency range is also known to provoke afterdischarges and was actually used in the kindling model of epilepsy (Goddard, 1967; Racine, 1972).

### High-frequency stimulation (HFS) condition

Finally, for the high-frequency stimulation (HFS, *f* > 70 Hz) condition, the direct and sustained excitation of reticular nucleus (RtN) interneurons leads to strong inhibition of *TC* cells and thus to dramatic decrease of their firing rate. Despite the fact that *TC* neurons are also affected by stimulation, the response of reticular GABAergic neurons to stimulation and the higher efficiency of GABA-mediated currents ensure that IPSPs override EPSPs on *TC* cells. In this case, the reduced excitatory input to cortical pyramidal cells also leads to a suppression of epileptic activity (Figure 9D). This result corroborates reported stimulation studies where HFS (>100 Hz) was associated with significant decrease in epileptiform discharges *in vitro*, and reduction in seizure frequency in responding patients (Velasco et al., 2006; Fisher et al., 2010). This hypothesis is in line with recent findings suggesting that HFS of the globus pallidus (GPi) in dystonia patients decreased its firing by stimulation-evoked GABA release from afferent fibers and thereby the enhancement of inhibitory synaptic transmission by HFS (Liu et al., 2012). Similarly, HFS (100–130 Hz) of the STN neurons in vitro showed a suppression of the activity of the majority of neurons by the reinforcement of inhibitory responses (Filali et al., 2004). Other HFS studies also provided evidence on the inhibition of GPi output during HFS in human patients (Dostrovsky et al., 2000) as well as the disruption thalamocortical network's dysrhythmia (McIntyre and Hahn, 2010; Kendall et al., 2011).

## Conclusion

In epilepsy research, it is well-admitted that there is, unfortunately, a lack of tangible results regarding the effects of electrical stimulation in the brain. Therefore, the very crucial issue of choosing the “optimal” stimulation parameters remains unsolved, whatever the stimulation procedure. Although computational models are always based on a number of simplifying assumptions, we think that they provide an efficient framework to (i) account for the many and essential factors that may intervene during stimulation procedures and (ii) analyze the links between these factors in a formal manner. This approach is particularly fruitful when models are well grounded in experimental/clinical data (Wendling et al., 2012). This is somehow a weak point of this study since we could make use of data sets recorded in one patient only. However, it should be mentioned that these very informative data sets stay relatively rare since many conditions have to be met (patient candidate to surgery, FCD, electrodes positioned in appropriate structures).

At this stage, the face value of the model is satisfactory. The next step is obviously to test the model predictions using animal models. Experiments can be undertaken in rodents with electrodes implanted in the cerebral cortex and in the thalamus. First, we could start with control animals to assess the modulation of cortical rhythms during/after direct thalamic stimulation at various frequencies and for controlled vigilance states (sleep, awake, resting, exploratory). In these controls, some drugs can be used to alter some parameters related to synaptic transmission (in a more or less specific manner) which have a correspondence in the model, on the other hand. Then, refined experimental models could be introduced to get closer to the epilepsy context including models of developmental dysplastic lesions [see review in Schwartzkroin and Wenzel (2012)]. Hopefully, this combined computational/experimental approach will help us to disclose some of the highly intricate effects of DBS either at local or at network level.

### Conflict of interest statement

The authors declare that the research was conducted in the absence of any commercial or financial relationships that could be construed as a potential conflict of interest.

## Abbreviations

CMN: Centromedian Nucleus
DBS: Deep Brain Stimulation
EPSP: Excitatory Postsynaptic Potentials
FCD: Focal Cortical Dysplasia
FFI: Feed-Forward Inhibition
GPi: Globus Pallidus
HFS: High Frequency Stimulation
iEEG: Intracerebral EEG (depth electrodes)
IFS: Intermediate Frequency Stimulation
IPSP: Inhibitory Postsynaptic Potentials
LFP: Local Field Potential
LFPs_FCD_: Local Field Potentials recorded in the FCD
LFS: Low Frequency Stimulation
NS: No Stimulation
PMC: Premotor cortex
RtN: Reticular thalamic Nucleus, STD, Short-Term Depression
STN: Subthalamic Nucleus.
